# Supplementary material for: Identifying long-range synaptic inputs using genetically encoded labels and volume electron microscopy
Source: Sci Rep. 2022 Jun 17;12:10213. doi: 10.1038/s41598-022-14309-4 (PMC9205864; doi:10.1038/s41598-022-14309-4)
Supplement: Supplementary file 1 — Supplementary Information. [file 41598_2022_14309_MOESM1_ESM.pdf]

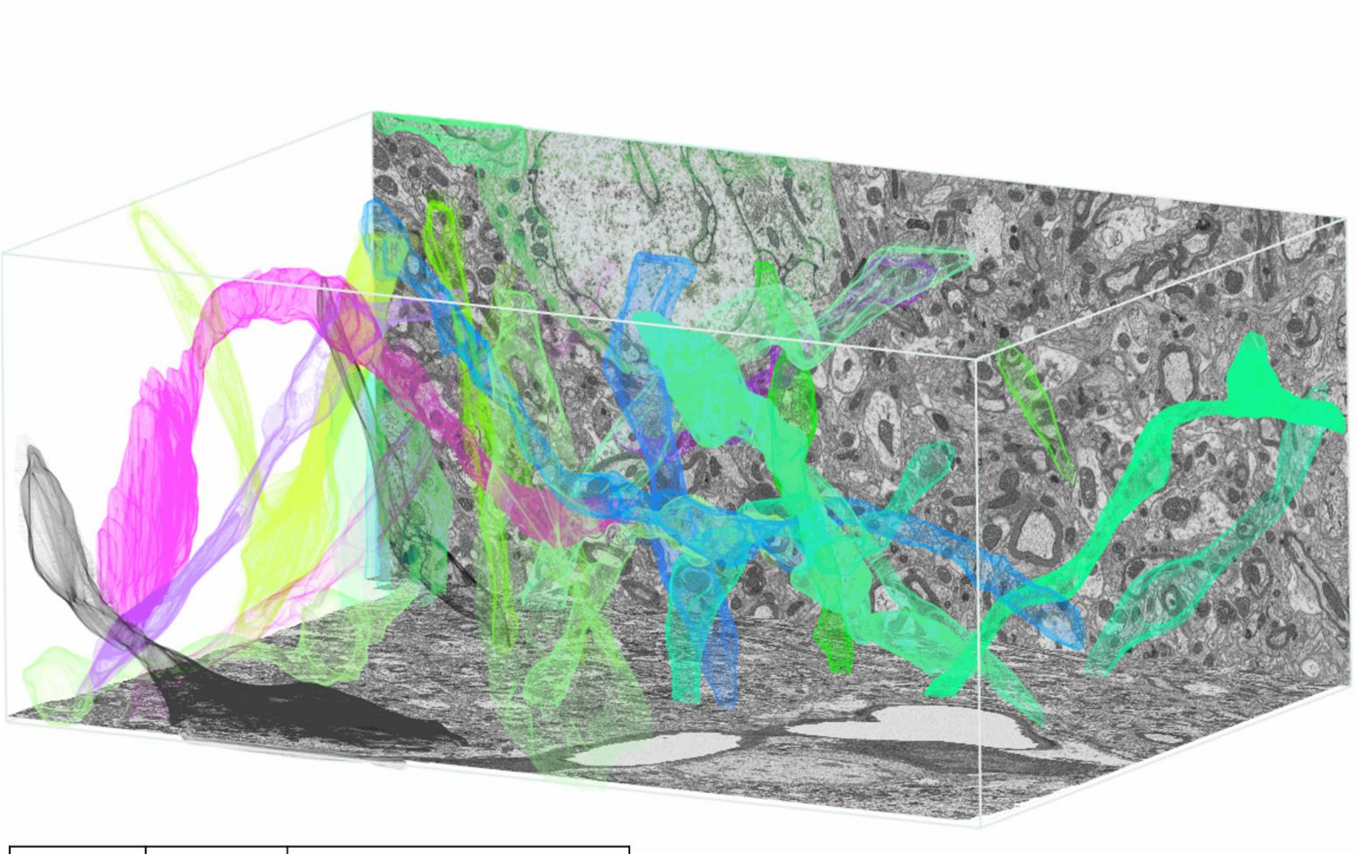

	With dendritic spines	Without dendritic spines
With ACC contacts	1,2,15,18	6, 20
Without ACC contacts	3,11	4, 5, 7, 8, 9, 10, 12, 13, 14, 15, 16, 17, 19, 21, 22, 23, 24

Supplementary Figure 1 | ER-stained dendrites are homogeneously distributed in a sample. Top: 3D reconstruction of 24 ER-dAPEX2 labeled dendrites across the entire sample volume (Sample 3). Bottom: most labeled dendrites were aspiny and did not receive ACC inputs.

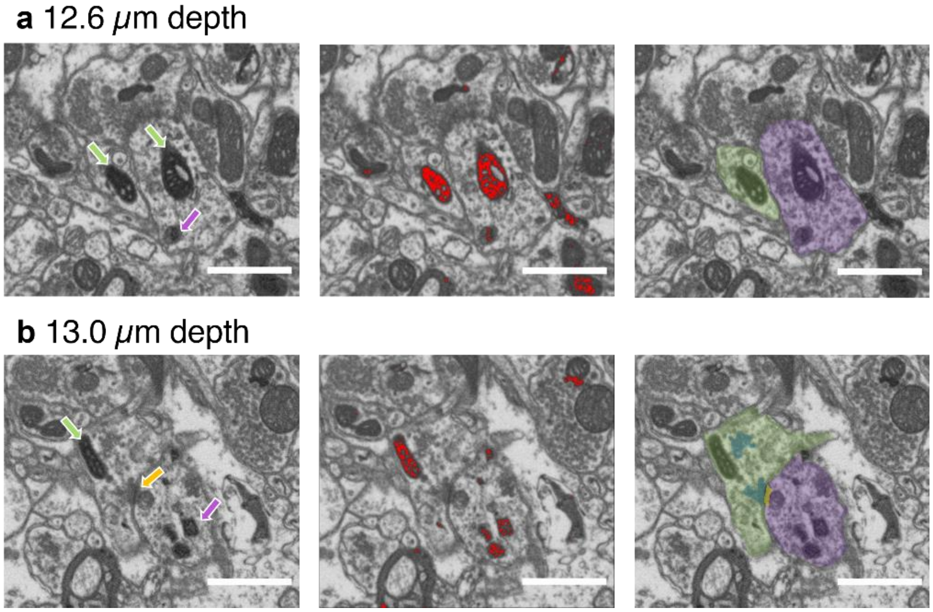

Supplementary Figure 2 | Matrix-dAPEX2 staining is occasionally observed in dendrites. a Left: Representative plane showing two labeled mitochondria (Sample 3). Middle: Same mitochondria are highlighted by binary mask. Right: Segmented process (green: axon; purple: dendrite). **b** Deeper plane of the same ROI showing synapse between Matrix-dApex2 labeled axon and Matrix-dApex2 labeled dendrite which also contains ER-dAPEX2 label (scale bar is 1 μm; green arrow: labeled mitochondrion; purple arrow: labeled ER yellow arrow: PSD).

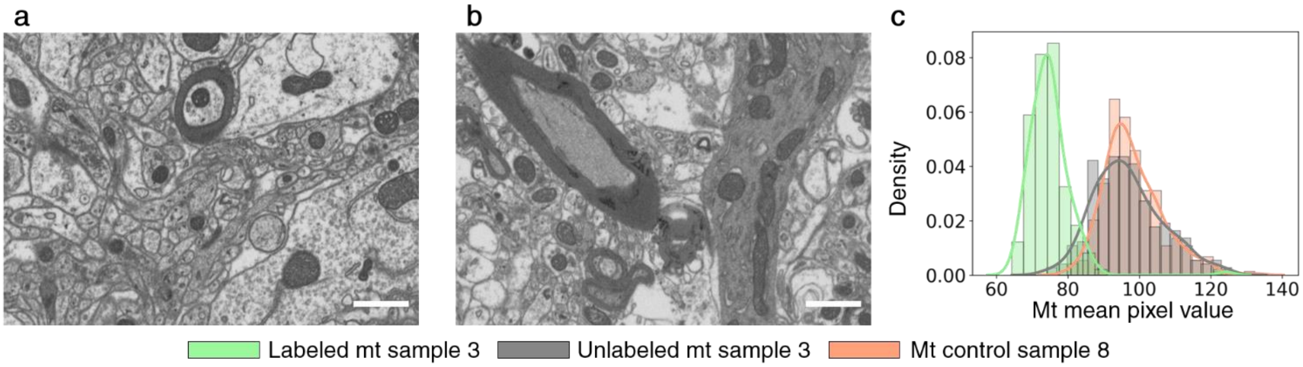

Supplementary Figure 3 | No Matrix or ER-dAPEX2 label found in control samples. Representative plane of control (Sample 7) showing satisfactory sample preparation quality and no dApex2 labeling. **b** Representative plane of control (Sample 8) showing satisfactory sample preparation quality and no dApex2 labeling. **c** Kernel density estimation and histogram of mitochondria mean pixel value selected from representatives XY and XZ planes in control (Sample 8) plotted with labeled and unlabeled mithocondria pixel values from a labeled sample (Sample 3; see **Fig. 1k**; scale bar is 1 μm).

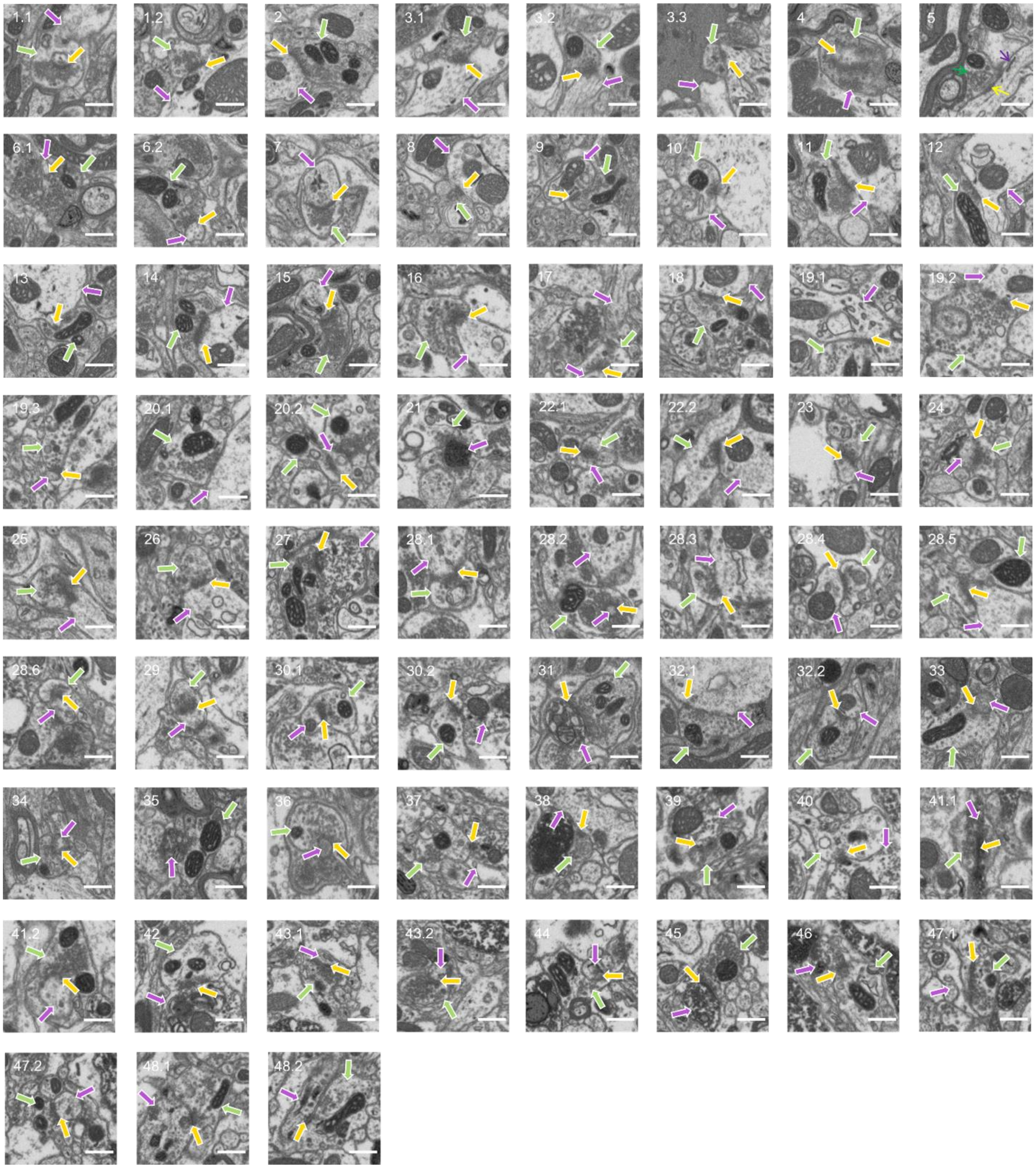

Supplementary Figure 4 | Library of sixty-seven analyzed ACC contact sites. (scale bar is 1 µm; green arrow: presynaptic cell with labeled mitochondrion; purple arrow: postsynaptic cell; yellow arrow: PSD).

Supplementary Table 1 | List of samples

**Sample ID**	**Animal ID, Sex**	**Animal genotype**	**Label configuration**	**Microscope acquisition**	**XYZ stack resolution**
Sample 1	5460, female	*Vglut2*::Cre	ACC: Matrix-dAPEX2<br>PAG: DIO-ER-APEX2	TEM	5x5 nm
Sample 2				SBEM	10x10x40 nm
Sample 3				FIBSEM	10x10x10 nm
Sample 4	5603, female				
Sample 5	7390, male				
Sample 6	7930, male	*Vgat*::Cre			
Sample 7	OBO-017266, male	*wild-type*	Control – no surgery		
Sample 8	8845, male	*Vglut2*::Cre			

Supplementary Table 2 | Summary of *Vglut2*+ dendrites and somas reconstructed in sample 3

Dendrite #	# Dendritic spines	# ACC contacts
1	1	2
2	3	1
3	1	0
4	0	0
5*	0	0
6*	0	1
7*	0	0
8	0	0
9	0	0
10	0	0
11	2	0
12*	0	0
13*	0	0
14	0	0
15	2	2
16	0	0
17	0	0
18	1	3
19	0	0
20	0	1
21	0	0
22	0	0
23	0	0
24*	0	0

*Neuron soma was present in the block
